# Comparison between single photon emission computed tomography with computed tomography and planar scintigraphy in sentinel node biopsy in breast cancer patients

**DOI:** 10.1007/s12149-018-1319-z

**Published:** 2018-11-19

**Authors:** Mitsuru Koizumi, Masamichi Koyama

**Affiliations:** Departments of Nuclear Medicine, Cancer Institute Hospital, 3-8-31 Ariake, Koto-ku, Tokyo, 135-8550 Japan

**Keywords:** Breast cancer, Radio-guided, Sentinel node detection, SPECT/CT, Planar scintigraphy, Contra-axilla visualization

## Abstract

**Objective:**

Radio-guided sentinel node (SN) biopsy is a standard method used in the treatment of early breast cancer. Single photon emission computed tomography with computed tomography (SPECT/CT) has been commonly used for SN detection. SPECT/CT adds precise anatomical information of SN sites, and it is reported that more SNs may be detectable on SPECT/CT than on planar imaging. We here investigate which breast cancer patients have benefited from SPECT/CT over planar imaging.

**Methods:**

A total of 273 breast cancer patients including 80 with ipsilateral breast tumor relapse (IBTR) underwent both multiple-view planar imaging and SPECT/CT for SN detection. The number of SNs, the patients who had benefitted from SPECT/CT, and the SN procedure failure rate were compared between SPECT/CT and planar imaging. Factors influencing the visualization of para-sternal and ipsilateral level II, III nodes, and contralateral axillary nodes were also analyzed using logistic regression analysis.

**Results:**

The number of hot spots did not differ between SPECT/CT and multiple-view planar imaging. Eight contaminated patients and 52 patients with visualized extra-level I axillary nodes benefited from identifying precise anatomical sites. Even though radioactive nodes could be harvested in most (192/193) of the non-IBTR patients (7/8 in non-SN visible patients), no radioactive nodes could be found during surgery in 11 of 80 IBTR patients. Axillary surgery (dissection) increased the visualization of para-sternal and level II, III axillary nodes, and previous irradiation increased the visualization of contralateral axillary nodes.

**Conclusion:**

Multiple-view planar imaging was equivalent to SPECT/CT for depicting hot nodes for radio-guided SN detection in breast cancer. SPECT/CT was useful when precise anatomical information was necessary, especially regarding sentinel lymph nodes other than ipsilateral axilla. Logistic regression analysis revealed that axillary surgery (dissection) increased the visualization of para-sternal and level II, III axillary nodes, and the only relevant factor influencing visualization of contralateral axillary SNs was previous radiation to the breast.

## Introduction

Radio-guided sentinel node (SN) biopsy is a standard method in the treatment of patients with early breast cancer [1–3]. Single photon emission computed tomography with computed tomography (SPECT/CT) has been used to detect hot nodes prior to surgery [4–9]. Recently, the EANM and SNMMI practice guideline for lymphoscintigraphy and sentinel node localization in breast cancer was published [10]. This states that the “current indication for SPECT/CT includes (1) non-visualization of SN on conventional planar imaging, (2) patient obesity, (3) the presence of extra-axillary SNs or otherwise difficult-to-characterize drainage (e.g., multiple sites of drainage, visualization of internal mammary node chain, intramammary lymph node, nodes in the contralateral axilla, or previous breast surgery). SPECT/CT might also be performed if the conventional images are difficult to interpret (e.g., suspicion of contamination or a SN near the injection area)”.

SPECT/CT has the advantages of better sensitivity and precise anatomical information, using a hybrid SPECT/CT machine and image fusion technique, over planar scintigraphy [4]. However, taking SPECT/CT increases procedure time and related patient discomfort and delivers a small but non-negligible radiation dose [7]. No universal method for radio-guided SN detection using a radiocolloid and scintigraphy currently exists [3, 10, 11]. However, re-injection of radiocolloid is reportedly more effective than the use of SPECT/CT [12]. We previously reported that taking multi-direction planar images improved the detection of SNs in breast cancer [13]. Patients who had been treated for ipsilateral breast cancer (ipsilateral breast tumor relapse; IBTR) were reported to frequently show unexpected extra-axillary drainage [14, 15]. Therefore, a method to identify the patients who would benefit from receiving SPECT/CT is needed.

In the present study, we aimed to clarify the added value of SPECT/CT over planar imaging for breast SN detection in our routine practice, and to determine which patient types are appropriate candidates for undergoing SPECT/CT. We also investigated the factors influencing the visualization of extra-ipsilateral level I axillary nodes and contralateral axillary nodes.

## Materials and methods

### Patients

Breast cancer patients who underwent breast surgery and SN detection with both multiple-view planar imaging and SPECT/CT from March 2015 to the end of August 2018 were retrospectively enrolled in this study. The initial inclusion criterion was that patients were randomly studied from March 2015 to October 2016. From November 2016, patients with ipsilateral breast surgery including IBTR, patients who were contaminated with radionuclide during the procedure, patients whose sentinel nodes were not detected on planar scintigraphic images, and patients who showed unexpected hot spots on planar images were selectively included in the study. Bilateral breast cancer patients were not included unless sequential imaging was performed [16]. The total number of patients was 273, including 80 patients with IBTR. Table 1 summarized the reasons why SPECT/CT was performed. Table 2 summarized patients’ previous treatments including breast surgery, axillary surgery, adjuvant therapy, and irradiation status of the IBTR patients.

**Table 1:** Reasons why SPECT/CT was performed

	Number of patients
No reason	148
Previous ipsilateral breast surgery	99
IBTR	80
Re-surgery for residual tumor	7
Benign tumor	6
Breast augmentation	6
Contamination	6
Non-visualization	9
Unexpected hot spot	11
PS	7
Contra-axilla	1
Rotter	1
intra-breast	2
Total	273

*IBTR* ipsilateral breast tumor relapse, *PS* para-sternal

**Table 2:** Previous treatments for patients with ipsilateral breast tumor relapse

	Number of patients
Breast surgery	
Partial	77
Total	3
Axillary nodes	
No	15
Sentinel node biopsy	52
Axillary dissection	13
Adjuvant therapy	
No	45
Hormone only	19
Chemotherapy	16
Breast irradiation	
No	49
Yes	31

This study was performed in accordance with ethical standards laid down in the 1964 Declaration of Helsinki and its later amendments and was approved by our local ethical committee. The need for informed consent was waived for this retrospective study.

### Sentinel node detection procedure

The sentinel node detection was carried out using a 2-day protocol, which involves the injection performed on the afternoon of the day before the surgery and the scintigraphy performed 1 h after the injection. Each patient received 55.5 MBq in 1 ml of Tc-99m phytate delivered peritumorally in each breast. The injection in each breast was given at four sites: two superficial and two deep injections [17]. One hour after the injection, planar images in 3 directions (anterior, 30° oblique, and 60° oblique views) were taken, and each scan time was 2 min with 512 × 512 matrices [13]. An additional lateral view was taken when no image was obtained on three views. The SPECT/CT study then followed, with 10 s’ data collection for each step, with 45 steps for 180 degrees of collection (dual cameras) with 128 × 128 matrices, and then, CT was performed to produce axial and coronal fusion images. Both planar imaging and SPECT/CT were performed using a Siemens Symbia Intevo16 (Siemens Healthcare) equipped with a low-energy high-resolution (LEHR) collimator.

### Sentinel node biopsy (SNB) procedure

A dye technique was also used in the surgery. Just prior to surgery, 2 ml of indigocarmine was injected under the areola. During the breast surgery, sentinel nodes were harvested using the dye technique and a gamma probe (Neoprobe, Devicor medical systems). The gamma count and extent of staining of each node were recorded. The harvested nodes were sent to the pathology unit, and rapid pathologic examination using frozen sections or a one-step nucleic acid amplification (OSNA) assay was performed during the surgery. A permanent pathological examination using hematoxylin eosin staining was performed after surgery. After obtaining rapid pathology or OSNA results, the surgeons decided whether axillary dissection should be performed.

### Analysis of data

The number of imaged hot nodes was compared between planar images and SPECT/CT images. When discrepancies existed, the surgical results were referred to. First, we judged the reproducibility or agreement between planar images and SPECT/CT using Wilcoxon rank sum test and Cohen’s κ values. Second, we selected the patients who were judged to have benefited from added SPECT/CT. Third, SNB failures (no hot node harvested during surgery) were investigated. A logistic regression analysis was performed with SNB failure as the dependent factor and previous breast surgery, previous axillary surgery, previous adjuvant therapy, and previous irradiation to the breast as independent factors. Fourth, the factors (previous breast surgery, previous axillary surgery, previous adjuvant therapy, and previous irradiation to the breast) influencing on the visualization of contralateral, and para-sternal and ipsilateral level II and III axillary sentinel nodes were explored using logistic regression analysis. Two analyses were conducted: one with para-sternal and ipsilateral level II and III node visualization as the dependent factors, and the other with contralateral axillary node visualization as the dependent factor. In both analyses, previous breast surgery, previous axillary surgery, previous adjuvant therapy, and previous irradiation to the breast were used as independent factors.

Statistical analysis software (SPSS version 24; IBM Corp., Armonk, NY, USA) was used. A value of *p* < 0.05 was considered to indicate statistical significance.

## Results

### Comparison of the number of axillary and extra-axillary hot nodes between planar imaging and SPECT/CT

**Fig. 1:**
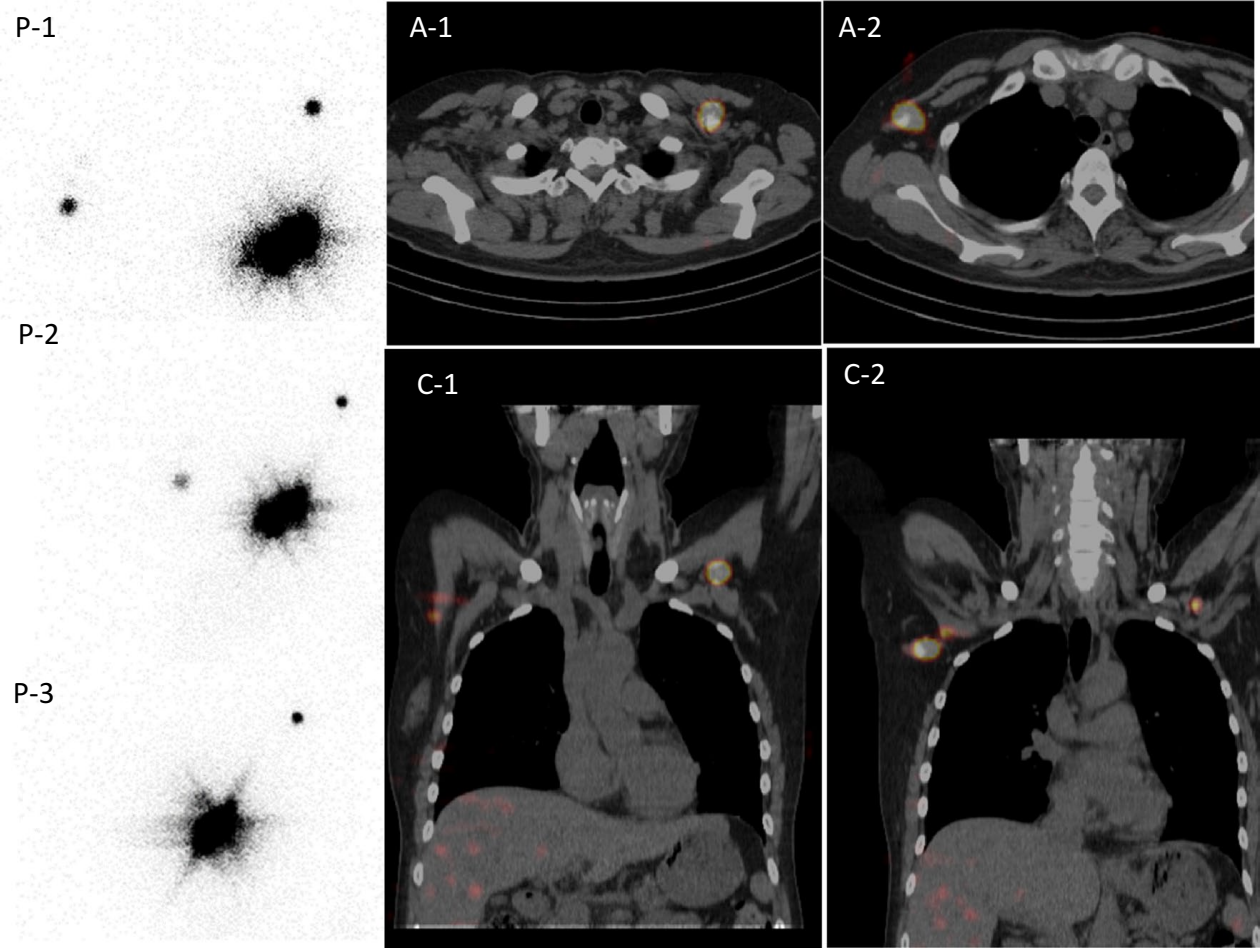
A 42-year-old woman received radio-guided sentinel node (SN) detection on both planar images and SPECT/CT images the day before left breast surgery for breast cancer. She had received ipsilateral breast surgery for breast cancer 3 years before; total mastectomy, SN biopsy followed by adjuvant tamoxifen therapy. Left breast recurrent tumor was diagnosed, and left breast re-surgery was planned. Planar images (P-1: anterior view, P-2: left anterior oblique, 30°, and P-3: left anterior oblique, 60°) and SPECT/CT (A-1,2: axial fusion images, C-1,2: coronal fusion images) had been shown. Planar image (P-1) illustrated contralateral axilla and ipsilateral axilla. SPECT/CT images clearly specified level I contralateral axilla and ipsilateral level II axilla (Rotter). On surgery, both lymph nodes were harvested and revealed to have cancer metastasis. Despite adjuvant hormone therapy after re-surgery, she developed local, lymph node, and distant metastasis 2 years later

Table 3 summarized the hot node detectability of planar imaging and SPECT/CT. The number of axillary node hot spots detected was concordant in 267 of 273 patients. Five patients showed one more sentinel node on SPECT/CT than on planar imaging, and one patient showed one more hot node on planar imaging than on SPECT/CT. In the five patients who showed one more hot node on SPECT/CT, the SPECT/CT results were concordant with the surgical results in two patients, planar results were concordant with the surgical results in two patients, and the concordance with the surgical results could not be judged in one patient. In the patient who showed one more hot node on planar imaging, the SPECT/CT result was concordant with the surgical result. Figure 1 showed a case whose planar images picked up more nodes than SPECT/CT. Two hot spots on planar images could not be separated using SPECT/CT; however, the precise anatomical information (Rotter) was of help in performing the surgical procedure.

**Table 3:** Comparison in number of hot spots between planar images and SPECT/CT

Detected nodes	Number of patients	Cohen’s *κ*^a^
Axillary nodes		0.967
		95% CI 0.942–0.992
Non-visualization	31	
Planar > SPECT/CT	1^b^	
Planar = SPECT/CT	236	
Planar < SPECT/CT	5^c^	
Extra-axillary nodes		1
		95% CI 1.00–1.00
Non-visualization	241	
Planar > SPECT/CT	0	
Planar = SPECT/CT	32	
Planar < SPECT/CT	0	

*CI* confidence interval

^a^Using the number of detected nodes on planar images and SPECT/CT, Cohen’ *κ* was calculated to evaluate the agreement between the two methods. There was an outstanding agreement between planar images and SPECT/CT

^b^SPECT/CT in this patient showed concordance with surgical results. In this patient, planar imaging showed 3 hot nodes and SPECT/CT showed 2 hot nodes. Two hot nodes were found during surgery

^c^In two patients SPECT/CT showed concordance with surgical results, in two patients planar imaging showed concordance with surgical results, and one patient could not be judged. Each patient showed one more hot node on SPECT/CT compared with planar images.

The number of extra-axillary hot nodes detected was concordant between planar imaging and SPECT/CT in all patients. Wilcoxon rank sum test showed no statistically significant differences between multiple planar imaging and SPECT/CT in the detection of either axillary (*p* = 0.102) or extra-axillary (*p* = 1.00) hot nodes. To investigate the concordance or agreement, the Cohen’s kappa (*κ*) coefficient was calculated as 0.967 [95% confidence interval (CI) 0.942–0.992] for axillary nodes and 1.00 (95% CI 1.00–1.00) for extra-axillary nodes. These figures were in perfect agreement.

### Patients for whom SPECT/CT added useful information

**Fig. 2:**
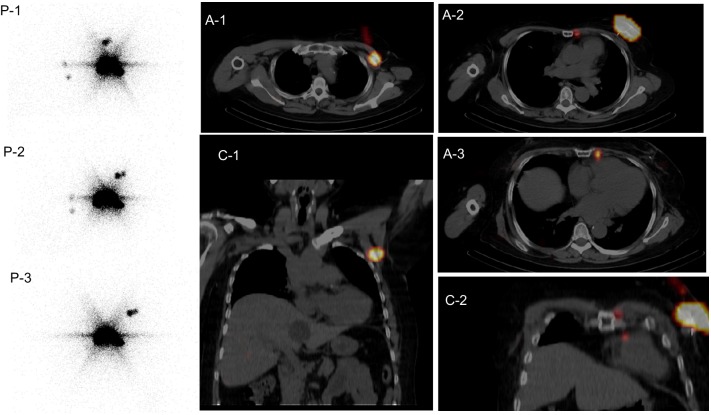
A 57-year-old woman received radio-guided sentinel node (SN) detection on both planar images and SPECT/CT images the day before left breast surgery for breast cancer. She had a long history, with right breast surgery (lumpectomy and axillary dissection) at 38 years of age, and left breast surgery (lumpectomy and axillary dissection) at 41 years of age. She developed new left breast cancer at 57 years of age. Planar images (P-1: anterior view, P-2: left anterior oblique, 30°, and P-3: left anterior oblique, 60°) and SPECT/CT (A-1,2,3: axial fusion images, C-1,2: coronal fusion images) had been shown. Planar image (P-1) illustrated two ipsilateral axillary nodes and two para-sternal nodes. SPECT/CT images specified one level II (Rotter) axillary node (A-1, C-1) and two para-sternal nodes (A-2,3, C-2). Left total mastectomy was performed and two Rotter nodes were harvested at surgery without cancer metastasis. Para-sternal nodes were not manipulated. She received adjuvant chemotherapy after surgery, and has remained without recurrence for 1 year

Table 4 summarized the patients for whom SPECT/CT added useful information over planar imaging. In eight patients who were contaminated during the injection procedure, the radioactivity contamination was clearly differentiated from true hot nodes. In 52 patients, anatomical information obtained from the SPECT/CT was helpful to search for hot nodes, especially nodes other than level I. Figure 2 illustrated a case who showed contralateral axilla nodes and para-sternal node, all of which were clearly imaged by both planar images and SPECT/CT. SPECT/CT added precise anatomical information.

**Table 4:** SPECT/CT added useful information over planar imaging

Reason	Number of patients	IBTR	Non-IBTR
Contamination	8	2	6
Site (anatomical information)			
Level II	11	6	5
Rotter	6	3	3
Level III	2	2	0
Para-sternal	14	6	8
Contra-axilla	17	16	1
Intra-breast	2	1	1
Total of sites	52		

*IBTR* ipsilateral breast tumor relapse

### Radio-guided SN detection and SNB failure

Table 5 summarized the radio-guided SNB procedure failures, in which no hot node was harvested during surgery. Nine of the non-IBTR patients showed non-visualization of a hot node on both planar imaging and SPECT/CT. However, hot nodes were detected using a gamma probe during surgery in 8 of these 9 patients, with only 1 patient showing no hot node.

**Table 5:** Sentinel node biopsy (SNB) failure

Imaged nodes	Number of patients	Hot node undetectable on surgery	SNB failure %^a^
Non-IBTR			
No image	9	1	0.5% (1/193)^b^
IBTR			
No image	9	8	
Only para-sternal node	3	2	13.8% (11/80)
Only contra-axillary node	9	1	

*IBTR* ipsilateral breast tumor relapse

^a^The percentage of SNB failures between non-IBTR and IBTR showed *p* < 0.001 by Fisher’s exact test

^b^The percentage of true failures should be low because SPECT/CT was performed in only a very small portion of all non-IBTR patients during the period

Nine of the IBTR patients showed non-visualization of a hot node on both planar imaging and SPECT/CT, and hot nodes were undetectable during surgery in 8 of them. Moreover, hot nodes were not harvested in 2 of 3 patients who showed only a para-sternal node on imaging, and 1 of 9 patients who showed only contralateral axillary nodes on imaging. In total, 11 of the 80 IBTR patients showed radio-guided SNB failure (13.8%). The percentage of SNB failures was much higher for IBTR than non-IBTR patients.

Table 6 showed the results of logistic regression analysis using SNB failure as the dependent factor. In univariate analysis, all independent factors had a statistically significant influence on SNB failure. However, multivariate analysis results showed that only previous radiation to the breast was a significant factor for increased risk of SNB failure.

**Table 6:** Results of the logistic regression analysis for SNB failure

Previous	Number of patients	Univariate			Multivariate (forward stepwise)^a^		
		*p* value	Odds	Odds (95% CI)	*p* value	Odds	Odds (95% CI)
Breast surgery							
No (base)	174						
Yes	99	0.002	23.862	3.053–186.510	0.074	7.985	0.816–78.146
Axillary node procedure							
No (base)	208						
Sentinel node	52	0.003	8.913	1.438–6.827	NS		
Axillary dissection	13	< 0.001	30.37	5.897–156.413			
Adjuvant therapy							
No (base)	234				NS		
Hormone therapy	20	0.167	3.139	0.620–15.895			
Chemotherapy	19	0.021	5.297	1.280–21.924			
Breast radiation							
No (base)	242	NS					
Yes	31	< 0.001	24.341	6.931–85.483	0.002	8.864	2.201–35.700

Logistic regression analysis was performed with SNB failure as a dependent variable

*CI* confidence interval, *NS* not significant

^a^Multivariate analysis was performed with a forward stepwise method with a likelihood ratio

### Factors influencing contralateral sentinel node visualization

The factors influencing contralateral axillary node and para-sternal node visualization were investigated using univariate and multivariate logistic regression analysis, as shown in Table 7 (dependent variable: para-sternal and ipsilateral level II and III node visualization) and Table 8 (dependent variable: contralateral node visualization). Table 6 showed that previous axillary surgery increased the odds [axillary dissection; 23.154 (95% CI 3.844–139.466)] of visualizing para-sternal and ipsilateral level II and III nodes, and previous irradiation to the breast decreased the odds [previous breast radiation; 0.158 (95% CI 0.031–0.807)]. Regarding contralateral node visualization (Table 7), breast surgery, axillary surgery, adjuvant therapy, and irradiation were significant factors according to the univariate analysis. However, only previous irradiation to the breast was a statistically significant factor by multivariate analysis. The odds for previous irradiation to the breast was 65.608 (95% CI 17.171–250.682). These results indicate that previous axillary surgery (dissection) may have increased the non-level I axillary node visualization; however, previous irradiation to the breast was the only factor that increased the visualization of contralateral axillary nodes.

**Table 7:** Results of the logistic regression analysis for para-sternal and ipsilateral level II and III axillary node visualization

Previous	Number of patients	Univariate			Multivariate (forward stepwise)^a^		
		*p* value	Odds	Odds (95% CI)	*p* value	Odds	Odds (95% CI)
Breast surgery							
No (base)	174				NS		
Yes	99	0.004	2.821	1.402–5.677			
Axillary node procedure							
No (base)	208						
Sentinel node	52	0.004	3.133	1.438–6.827	< 0.001	4.366	1.932–9866
Axillary dissection	13	0.004	5.875	1.754–19.676	0.001	23.154	3.844–139.466
Adjuvant therapy							
No (base)	234				NS		
Hormone therapy	20	0.035	3.03	1.079–8.506			
Chemotherapy	19	0.669	1.325	0.364–4.829			
Breast radiation							
No (base)	242	NS					
Yes	31	0.862	0.906	0.298–2.753	0.027	0.158	0.031–0.807

Logistic regression analysis was performed with para-sternal and ipsilateral level II and III axillary node visualization as a dependent variable

*CI* confidence interval, *NS* not significant

^a^Multivariate analysis was performed with a forward stepwise method with a likelihood ratio

**Table 8:** Results of the logistic regression analysis for contra-axillary visualization

Previous	Number of patients	Univariate			Multivariate (forward stepwise)^a^		
		*p* value	Odds	Odds (95% CI)	*p* value	Odds	Odds (95% CI)
Breast surgery							
No (base)	174				NS		
Yes	99	0.001	33.349	4.349–255.751			
Axillary node procedure							
No (base)	208						
Sentinel node	52	0.001	10.63	2.646–42.696	NS		
Axillary dissection	13	< 0.001	79.755	16.463–386.05			
Adjuvant therapy							
No (base)	234				NS		
Hormone therapy	20	< 0.001	13.898	4.117–46.919			
Chemotherapy	19	0.002	8.648	2.276–32.86			
Breast radiation							
No (base)	242						
Yes	31	< 0.001	65.608	17.171–250.682	< 0.001	65.608	17.171–250.682

Logistic regression analysis was performed with contra-axillary node visualization as a dependent variable

*CI* confidence interval, *NS* not significant

^a^Multivariate analysis was performed with a forward stepwise methods with a likelihood ratio

## Discussion

Breast cancer is the most common malignancy in women. Lymph node status is of importance for determining prognosis. Regional staging was previously based on regional lymph node dissection and histopathological assessment. This method was accompanied by high risks of lymphedema, infections, impairment of sensitivity, and morbidity. In recent years, SNB has been considered the gold standard. This minimally invasive method evaluates the regional draining basins originating from primary tumor sites. If the SN does not contain metastasis, the remaining nodes have a negligible chance of having malignant cells. The technique begins with planar scintigraphy. SPECT/CT has been used to improve the detection of hot nodes and obtain precise anatomical information prior to surgery. Therefore, we aimed to clarify the added value of SPECT/CT over planar imaging for breast SN detection in our routine practice, and to determine which kinds of patient are appropriate candidates for undergoing SPECT/CT. We also investigated the factors influencing the visualization of extra-ipsilateral level I axillary nodes and contralateral axillary nodes.

Contrary to the reported results [4–9], we did not find a difference in the number of hot nodes identified using planar imaging and SPECT/CT. There were no patients whose SNs were visualized only on SPECT/CT and not on planar images. Even though the actual reasons for this discrepancy could not be determined, we speculated that the following could be responsible: (1) the radiocolloid was ^99m^Tc-phytate, (2) the injection sites including both superficial and deep sites, and (3) multiple-view (3–4) planar imaging was employed in each patient. ^99m^Tc-phytate is a unique colloid with small particles (8 nm) pre-injection, with colloid formation (100–200 nm) beginning after administration with exposure to calcium in the blood [18]. We previously reported that different injection sites resulted in different visualization of SNs [17]. The combination of both superficial and deep injection sites may have contributed to our result. The use of multiple-direction views may also have contributed to our result, as hot nodes that may have been hidden in one view may be shown in another [13].

In most patients other than those with IBTR, hot nodes could be harvested during surgery, even though SNs were not visible on both planar imaging and SPECT/CT. However, in patients with IBTR and showing no SN visualization, radioactive nodes were often not found during surgery. The results are concordant with previous findings [15]. Improved methods, for example, re-injection [7] might be needed in patients with IBTR.

By obtaining precise anatomical information from hybrid SPECT/CT, the patients who were contaminated with radionuclide could be clearly judged on SPECT/CT (especially fusion images). In cases of extra-axillary hot spots, precise anatomical information from the SPECT/CT fusion images provided useful information for the surgeons. Such information can help surgeons make a better surgical plan. Our logistic regression analysis (Table 6) showed that previous radiation to the breast increased the risk of failure. This may indicate that the reduced elasticity of lymphatic vessels by previous radiation caused reduced lymphatic flow and resulted in SNB failure.

SPECT/CT increases procedure time with related patient discomfort and delivers a small but non-negligible radiation dose. Therefore, we now add SPECT/CT when contamination is suspected on planar imaging, unexpected (extra-level I axillary) nodes are suspected on planar imaging, and for IBTR patients. We also used logistic regression analysis to study the factors that may be relevant to para-sternal and ipsilateral level II and III node visualization and contralateral axillary. Axillary surgery (dissection) increased the visualization of para-sternal and ipsilateral level II, III axillary nodes (Table 7), and previous irradiation increased the visualization of contralateral axillary nodes (Table 8). We speculate that axillary surgery (dissection) influenced or increased the lymphatic flow other than level I axilla; however, previous radiation to the breast was the only factor that increased the odds of visualization of contralateral axillary nodes.

The major drawback of the present investigation was that it was a retrospective single center study. Therefore, in the respect that we did not find a difference in the number of hot nodes between planar imaging with multiple projections and SPECT/CT, the interpretation of logistic regression analysis must await further judgment. The numbers of events were not sufficient; therefore, in the logistic regression analysis, we investigated only four independent factors. The results may change when a larger number of patients is investigated.

## Conclusions

We compared radio-guided SN detection between multiple-view planar imaging and SPECT/CT in breast cancer patients. The number of SNs detected did not differ between the two methods. The precise anatomical information obtained by SPECT/CT was useful in contaminated patients and for SNs other than level I nodes. The logistic regression analysis revealed that previous axillary dissection increased the visualization of para-sternal nodes and level II, III axillary nodes, and previous radiation to the breast increased the visualization of contralateral axillary nodes.

## References

[1] Krag D, Weaver D, Ashikaga T, Moffat F, Kimberg VS, Shiriver C (1998). The sentinel node in breast cancer—a multicenter validation study. N Engl J Med.

[2] Veronesi U, Paganelli G, Viale G, Luini A, Zurrida S, Galimberti V (2003). A randomized comparison of sentinel-node biopsy with routine axillary dissection in breast cancer. N Engl J Med.

[3] Koizumi M, Nomura E, Yamada Y, Takiguchi T, Makita M, Iwase T (2004). Radioguided sentinel node detection in breast cancer patients: comparison of 99mTc phytate and 99mTc rhenium colloid efficacy. Nucl Med Commun.

[4] Keidar Z, Israel O, Krausz Y (2003). SPECT/CT in tumor imaging: technical aspects and clinical applications. Sem Nucl Med.

[5] Lerman H, Metser U, Lievshitz G, Sperber F, Shneebaum S, Even-Sapir E (2006). Lymphoscintigraphic sentinel node identification in patients with breast cancer: the role of SPECT/CT. Eur J Nucl Med Mol Imaging.

[6] Borrelli P, Donswijk ML, Stokkel MP, Teixeira SC, van Tinteren H, Rutgers EJ (2017). Contribution of SPECT/CT for sentinel node localization in patients with ipsilateral breast cancer relapse. Eur J Nucl Med Mol Imaging.

[7] Jimenez-Heffernan A, Ellmann A, Sado H, Huic D, Bal C, Parameswaran R (2015). Results of a prospective multicenter international atomic energy agency sentinel node trial on the value of SPECT/CT over planar imaging in various malignancies. J Nucl Med.

[8] Valdes Olmos RA, Vidal-Sicart S, Manca G, Mariani G, Leon-Ramirez LF, Rubello D (2017). Advances in radioguided surgery in oncology. Q J Nucl Med Mol Imaging.

[9] Kraft O, Havel M (2013). Sentinel lymph nodes and planar scintigraphy and SPECT/CT in various types of tumours. Estimation of some factors influencing detection success. Nucl Med Rev.

[10] Giammarile F, Alazraki N, Aarsvold JN, Audisio RA, Glass E, Grant SF (2017). The EANM and SNMMI practice guideline for lymphoscintigraphy and sentinel node localization in breast cancer. Eur J Nucl Med Mol Imaging.

[11] Simanek M, Koranda P (2016). SPECT/CT imaging in breast cancer—current status and challenges. Biomed Pap Med Fac Univ Palacky Olomouc Czech Repub.

[12] Pouw B, Hellingman D, Kieft M, Vogel WV, van Os KJ, Rutgers EJ (2016). The hidden sentinel node in breast cancer: reevaluating the role of SPECT/CT and tracer reinjection. Eur J Surg Oncol.

[13] Koizumi M, Nomura E, Yamada Y, Takiguchi T, Ishii M, Yamashita T (2004). Improved detection of axillary hot nodes in lymphoscintigraphy in breast cancer located in the upper lateral quadrant with additional projection imaging. Ann Nucl Med.

[14] Koizumi M, Koyama M, Tada K, Nishimura S, Miyagi Y, Makita M (2008). The feasibility of sentinel node biopsy in the previously treated breast. Eur J Surg Oncol.

[15] van der Ploeg IM, Oldenburg HS, Rutgers EJ, Baas-Vrancken Peeters MJ, Kroon BB, Valdes Olmos RA (2010). Lymphatic drainage patterns from the treated breast. Ann Surg Oncol.

[16] Koizumi M, Koyama M, Morizono H, Miyagi Y (2018). Sequential sentinel node scintigraphy with planar and SPECT/CT images revealed contralateral drainage from ipsilateral breast tumor relapse in a patient with bilateral breast cancer. Clin Nucl Med.

[17] Koizumi M, Koyama M, Yamashita T, Tada K, Nishimura S, Takahashi K (2006). Experience with intradermal injection and intradermal-plus-deep injection in the radioguided sentinel node biopsy of early breast cancer patients. Eur J Surg Oncol.

[18] Cho Y, Lee K (2017). Feasibility of 99mTc-phytate as a lower lymphoscintigraphic agent in dogs. Pak Vet J.

